# Biomarker-Based Diagnosis of Contact Dermatitis: A Step Towards More Accurate and Patient-Friendly Testing

**DOI:** 10.3390/clinpract15120217

**Published:** 2025-11-21

**Authors:** Nique Grob, Thomas Rustemeyer, Florentine de Boer

**Affiliations:** 1 Department of Dermatology and Allergology, Vrije Universiteit Amsterdam, 1081 BT Amsterdam, The Netherlands; 2 Department of Dermatology and Allergology, Amsterdam UMC, 1105 AZ Amsterdam, The Netherlands

**Keywords:** contact dermatitis, biomarkers, cholesterol sulfate, cholesterol glucosyl, non-invasive diagnostics

## Abstract

Background: Contact dermatitis (CD) is a prevalent inflammatory skin condition, with diagnostic challenges in distinguishing allergic (ACD) from irritant contact dermatitis (ICD). This study aimed to explore cholesterol-derived biomarkers as potential diagnostic tools. As cholesterol derivatives play key roles in skin barrier integrity and inflammation, they are promising candidates for assessing skin barrier disruption in CD. Methods: Stratum corneum samples were collected by tape stripping from experimentally induced and chronic lesions, as well as healthy non-lesional skin. Biomarkers Cholesterol Sulfate (Chol-Sulf), Cholesterol Glucosyl (Chol-Glc) and their ratio were quantified. Data were analyzed using ANOVA, Pearson’s correlation, logistic regression and ROC curves. Results: Chol-Glc and the Chol-Glc/Chol-Sulf ratio differed significantly across the diagnostic groups, while Chol-Sulf did not. Logistic regression and ROC analyses revealed a limited standalone diagnostic accuracy for the individual biomarkers (all AUC < 0.6). Conclusions: Chol-Glc and the ratio exhibit disease-specific patterns relevant for subtype discrimination. Although insufficient as independent diagnostic tools, these markers may contribute to future multivariate diagnostic models for CD diagnosis.

## 1. Introduction

Contact dermatitis (CD) is a highly prevalent inflammatory skin disorder, affecting at least 20% of the general population [1]. In addition to its physical burden of dry and scaly skin, CD carries a considerable mental burden, as its visible symptoms are often paired with embarrassment, self-consciousness, depression, social withdrawal and reduced work productivity [2,3,4]. Given this broad impact on both physical and mental well-being, achieving an accurate and rapid diagnosis is essential for effective treatment, thereby reducing visible symptoms and improving the patient’s quality of life.

The two main types of CD are irritant contact dermatitis (ICD) and allergic contact dermatitis (ACD) [5]. ICD results from a repeated or prolonged exposure to irritants such as soaps and detergents, causing direct damage to the skin barrier [6]. This damage activates the innate immune system, leading to the release of pro-inflammatory cytokines and a resulting inflammatory cutaneous response [5,6]. This inflammatory response can in turn lead to a loss of structural proteins and lipids, thereby weakening the epithelial barrier [7]. This disruption increases transepidermal water loss (TEWL), which leads to the dry scaly skin seen in CD, as well as a rise in the permeability of the skin, thus rendering it more susceptible to irritants [7].

Unlike ICD, ACD is an adaptive immune-mediated disorder which consists of two phases. During the sensitization stage, the skin is exposed to small chemicals known as haptens, which penetrate the skin and bind to self-proteins. These cells then present the antigens to naïve T cells in lymph nodes, rendering a person sensitized to them due to immunological memory formation [8]. This means that the immune system is now primed to have a higher and faster response when re-exposed to the allergen in the elicitation stage [8]. During this stage, a delayed type four hypersensitivity reaction takes place 24–72 h after the exposure, which results in inflammation and epidermal damage, leading to an eczematous skin reaction [9,10]. This response can already be triggered by small amounts of an allergen in sensitized individuals, unlike ICD, where a higher concentration and longer exposure to irritants are typically required.

The pathophysiology of ICD and ACD shows a clear divide between the two; although both involve immune mechanisms, ICD is caused by direct barrier disruption, while ACD results from delayed immune activation. However, their clinical presentation is very similar, making differentiation between them difficult [10]. Currently, the golden standard for distinguishing ICD from ACD is an epicutaneous patch test, which assesses delayed hypersensitivity responses, rendering it able to detect or rule out ACD [11]. A negative patch test reaction, in combination with clinical patient history and exposure profiles, is used to support an ICD diagnosis. However, this patch test has several limitations, the first of which is that a positive test does not necessarily implicate the allergen as the cause of the currently observed eczema. The patient may be sensitized to multiple allergens or irritants, complicating diagnoses via this test [12]. Moreover, the interpretation of the test is not standardized but subjective, which increases the risk of both false-positive and false-negative outcomes [13].

Lastly, the patch test can be a burden for the patient, as there are restrictions in daily life such as the avoidance of water, sweating and physical activity for multiple days, as well as potential discomfort from itching or irritation at the test site [14]. Given these challenges, there is an unmet need for more novel approaches that may improve differentiation between ICD and ACD in clinical practice.

**Figure 1:**
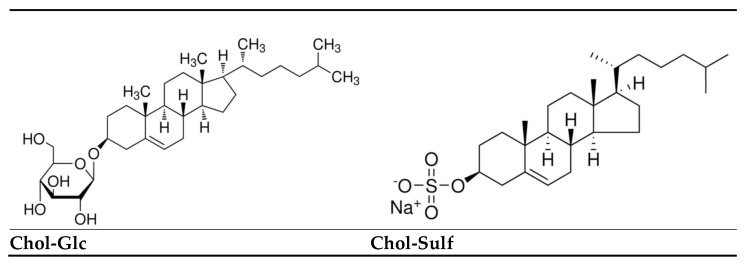
Chemical structures of Cholesterol Glucoside (Chol-Glc, **left**) and Cholesterol Sulfate (Chol-Sulf, **right**). Both are cholesterol-derived lipids differing in their polar head groups: Chol-Glc contains a β-D-glucosyl moiety at the C3 hydroxyl, whereas Chol-Sulf carries a sulfate ester group at the same position. These structural differences influence their polarity, epidermal distribution and respective roles in immune signaling and barrier organization. Images adopted from Sigma-Aldrich [18].

In order to close this gap, this study explores the biomarker patterns of Cholesterol Sulfate (Chol-Sulf), Cholesterol Glucosyl (Chol-Glc) and their ratio (Chol-Glc/Chol-Sulf) in both ACD and ICD to assess their potential for distinguishing between the two, thus evaluating their potential utility as diagnostic tools. These particular cholesterol derivatives were selected for this purpose as they play distinct roles in skin physiology and immune responses. Chol-Sulf regulates lipid structure, desquamation and keratinocyte differentiation, all of which are of great importance for preserving epidermal barrier integrity [15,16]. In contrast, Chol-Glc is involved in Th2-driven inflammatory responses and immune cell signaling pathways in deeper epidermal layers [17]. The cholesterol derivatives differ in their polar head groups. These structural differences influence their lipid organizational and signaling functions, relevant to epidermal barrier preservation. These structural changes can be seen in Figure 1.

The rationale for exploring Chol-Glc is supported by previous research, showing elevated levels of Chol-Glc in inflammatory skin conditions such as atopic dermatitis (AD) [19]. While AD is pathophysiologically different from CD, the association between Chol-Glc and inflammatory skin disorders is a cause for the exploration of this biomarker in the context of CD. The biomarkers’ unique roles within skin physiology suggest their potential role as indicators of both barrier disruption and immune activation, which is seen in CD. Rather than testing a fixed hypothesis, this study aimed to explore the patterns of the biomarkers across ACD and ICD in the hopes of identifying patterns that support the development of more objective, accurate and patient-friendly diagnostic alternatives to the patch test. Altogether, the central aim of this study is to answer the following question: do Chol-Sulf and Chol-Glc reflect distinct biomarker patterns in ICD and ACD that support their potential use in future diagnostic applications?

To enable the exploration of these biomarker patterns, patients with contact dermatitis were subjected to patch tests with various allergens and an irritant, which were subsequently analyzed for Chol-Sulf and Chol-Glc levels, as well as their ratio. The applied substances were selected to ensure the applicability of the findings to real-life settings, as they all have established roles in occupational CD, reflecting common exposures encountered in construction, manufacturing and healthcare environments [20,21,22,23]. An analysis of the samples may reveal distinct biomarker patterns associated with each condition, which could in turn be used for differentiating between ICD and ACD, possibly leading to faster diagnoses. If this proves to be validated, these findings could introduce a novel, biomarker-based diagnostic tool, enabling earlier intervention, faster recovery and improved quality of life for CD patients.

## 2. Materials and Methods

### 2.1. Experimental Design and Setting

This observational, cross-sectional, secondary analysis was conducted at and based on an existing dataset from the Occupational Dermatology and Allergology Clinic of Amsterdam UMC. The aim was to explore the previously collected data profiles of Chol-Sulf, Chol-Glc and their ratio within ICD and ACD to evaluate their potential for future diagnostic use in distinguishing between the two conditions. To capture both acute and chronic inflammatory states, stratum corneum samples were obtained from experimentally induced ICD and ACD via patch testing, as well as from chronic lesions on the hands of the same patient population via tape stripping. Samples from a patch test with petrolatum served as the control for patch data, where non-lesional hand skin of these patients was introduced as the control for biomarker levels. Ethical review and approval were waived under Dutch non-WMO regulations for this secondary analysis, as no new interventions or participant contact occurred. Moreover, all participants provided written, informed consent for collection and data use.

### 2.2. Participants

A total of 205 adult patients aged 18 to 65 years presenting with CD were initially involved. Inclusion criteria for further analysis included a positive patch test for one of the investigated allergens or irritants, a clinical diagnosis of either ACD or ICD and written informed consent. Clinical diagnoses were established based on patch test results, clinical symptoms and exposure history. Exclusion criteria were topical corticosteroid use within 24 h prior to sampling, recent systemic anti-inflammatory treatment, antibiotic use, phototherapy, pregnancy, lactation and trauma or active infection at the test site. After applying these criteria, 118 patients remained eligible for further analysis.

### 2.3. Sampling of Tape Strips

All clinical procedures, including patch testing, sample collection and laboratory analyses, were carried out by the research team at Amsterdam UMC prior to data handover. An anonymized dataset containing the processed biomarker values, expressed in picomoles per milligram (pmol/mg), as well as metadata was used for all subsequent statistical analyses in this study.

Each participant underwent standardized patch testing using a panel of occupationally relevant allergens and an irritant. These included nickel, chromium, methylisothiazolinone/methylchloroisothiazolinone (MI/MCI) and epoxy resin as allergens and 2% aqueous sodium lauryl sulfate (SLS) as the irritant. Patches were applied using Finn Chambers (SmartPractice, Phoenix, AZ, USA) and evaluated after 48 and 72 h in accordance with ESCD guidelines [24]. Following the patch testing, stratum corneum samples were collected via tape stripping from three skin sites per participant: a patch test site with a confirmed positive reaction, a chronic lesion site and a non-lesional control site. Tape stripping was performed using 14 mm D-Squame discs (CuDerm, Dallas, TX, USA) under standardized pressure. Ten consecutive strips were collected per patient and stored at −80 °C until analysis.

For the analysis of the cholesterol biomarkers, primarily the 8th tape strip was used. This decision was based on the existing literature, which guided the selection of specific tape strips for different analytical purposes. Strip eight was considered to be the most suitable for cholesterol analysis, while the other strips were allocated for additional investigations, such as for cytokines and natural moisturizing factors (NMFs).

### 2.4. Data Preparation and Sample Classification

Following data handover, group harmonization and statistical analyses were performed. Initially, samples were labeled based on the specific test substance applied, as well as the anatomical sampling site and lesion status. For analytical purposes, these samples were regrouped into five diagnostic categories: all allergen-induced sites were merged into one group, which was labeled ‘ACD induced’ (ACDi), and SLS-induced sites were labeled ‘ICD induced’ (ICDi). Chronic lesions on the hands were split based on clinical diagnosis and classified as chronic allergic lesions (L-ACD) or chronic irritant lesions (L-ICD), where clinically unaffected skin was labeled non-lesional (NL). Samples were grouped based on diagnosis rather than by individual test substance to enable a focused and clear comparison between ACD and ICD. This approach aimed to facilitate a robust statistical analysis and identify biomarker patterns specific to the underlying pathophysiology of each condition.

### 2.5. Statistical Analysis

To determine whether parametric or non-parametric methods were appropriate, the distributions of Chol-Glc, Chol-Sulf and their ratio were assessed visually using histogram plots with overlaid normality curves to evaluate symmetry and normality. Visual inspection via histograms was chosen over formal normality tests, as such tests can be overly sensitive and likely to indicate significant deviations from normality, even with minor deviations in large datasets [25].

All three variables showed right-skewed distributions in their original form and thus deviations from normality. Therefore, to assess whether transformation could improve normality, a log-transformation was applied to all three biomarkers, generating three new variables: log_Chol-Glc, log_Chol-Sulf and log-Ratio. These new variables were subsequently re-evaluated using the same visual inspection method. The log-transformation resulted in noticeably more symmetric distributions across all three variables, with all complying with the normality curve. Based on these visual assessments, all three newly generated biomarkers were analyzed using parametric statistical methods. Parametric tests were preferred, as they offer greater statistical power and easier interpretation than non-parametric tests; however, they could only be applied after the variables proved to comply with normality [26].

To investigate whether general alterations in the cholesterol markers were associated with CD, separate one-way ANOVAs were performed across NL, ACDi and ICDi for each log-transformed biomarker variable. To facilitate this comparison, a new numeric grouping variable, groepscode_clean, was created by recoding the original diagnostic group variable, ‘grouphernoemd’. Samples labeled as NL, ACDi or ICDi were assigned values 1, 2 and 3, respectively, while samples from chronic lesions (L-ACD, L-ICD) were excluded by assigning them a system-missing value. This ensured that the ANOVA only included the induced diagnostic groups relevant for this analysis, as chronic lesions are influenced by a multitude of factors and time, making them less directly comparable to the patch test-induced sites.

If the ANOVA indicates a significant main effect (*p* < 0.05), this means that at least one group median significantly differs from the others, prompting pairwise post hoc Tukey tests for the following group comparisons: ACDi vs. NL, ICDi vs. NL (non-lesional) and ACDi vs. ICDi. These comparisons help clarify whether each biomarker can distinguish not only diseased from healthy skin, but also between the two CD subtypes. This step was essential to determine whether a given biomarker is specifically altered between two diagnostic groups, or whether multiple groups differ from each other in distinct ways. The Tukey post hoc test was specifically chosen, as it reduces the risk of type-1 errors while maintaining statistical power when performing multiple group comparisons [27].

Next, Pearson’s correlations were used to see whether Chol-Glc and Chol-Sulf are co-regulated or rise and fall independently. This correlation was carried out both across the full dataset and within each diagnostic subgroup. A strong correlation implies that the two biomarkers overlap, thereby providing similar information, potentially rendering combined use redundant. However, weak correlations may indicate that each marker provides unique information, which could enhance their diagnostic utility when used in combination. For interpretation, correlation coefficients (r) were categorized as very weak (<0.20), weak (0.20–0.39), moderate (0.40–0.59), strong (0.60–0.79) or very strong (≥0.80). These interpretation thresholds are based on conventional guidelines for biomedical research [28].

Lastly, a logistic regression model was constructed to assess the ability of each biomarker to distinguish between ACDi and ICDi. No covariates were added to the model in order to allow for a clear evaluation of each biomarker’s standalone diagnostic value. Odds ratios (ORs) with 90% confidence intervals were reported to quantify the strength of the association between each biomarker and the diagnosis. An OR > 1 indicates that higher levels of the marker are associated with a greater likelihood of that diagnosis, while an OR < 1 suggests that higher levels reduce the likelihood of that diagnosis. The 90% confidence interval was applied to allow for the better detection of possible effects. While an OR can reflect association strength, it is not capable of reflecting the predictive value of the biomarkers. Therefore, predicted probabilities were saved, enabling the assessment of each biomarker and its diagnostic performance. These probabilities range from 0 to 1 and indicate the estimated likelihood for ACDi, with ACDi coded as 1 and ICDi coded as 0. To further assess the diagnostic performance of each biomarker, these predicted probabilities were used to construct receiver operating characteristic (ROC) curve analyses to visualize diagnostic accuracy. Since ROC analysis requires binary classification, three separate pairwise group comparisons were performed: ACDi vs. ICDi, ACDi vs. NL and ICDi vs. NL. For each comparison, the area under the ROC curve (AUC) was calculated as a summary measure of diagnostic accuracy. AUC values closer to 1 indicate a strong discriminatory ability, while an AUC value of 0.5 suggests no better predictive ability beyond chance. All analyses were performed using IBM SPSS Statistics version 30 (IBM Corp., Armonk, NY, USA). A *p*-value < 0.05 was determined to be statistically significant.

## 3. Results

### 3.1. Participant Demographics

The analyses were conducted for the cholesterol levels of 118 patients, aged between 18 and 65 years (mean age = 42.4 years, standard deviation (SD) = 16.8). Of these patients, 80 were female and 29 were male, with the sex information unavailable for the remaining 9 participants. Biomarker levels were examined in the following diagnostic groups: NL (n = 105), ACDi (n = 88), ICDi (n = 29), L-ACD (n = 25) and L-ICD (n = 13). Patients with diagnoses of more than one type of CD were excluded from the analyses.

### 3.2. Group Comparisons of Biomarker Levels (ANOVA)

Log-transformed biomarker levels were first compared using one-way ANOVAs across diagnostic groups, including non-lesional skin (NL), allergic contact dermatitis (ACDi) and irritant contact dermatitis (ICDi). Mean Chol-Sulf levels were lowest in the NL group (0.41 ± 0.32), followed by ICDi (0.51 ± 0.19) and then ACDi (0.55 ± 0.24). Levene’s test confirmed the homogeneity of variances (*p* = 0.185), validating the comparison of the groups. Moreover, a significant group effect was found (*p* = 0.002), with a small effect size (η^2^ = 0.055), meaning that the diagnostic groups do significantly differ from each other in Chol-Sulf values; however, this difference is not substantial. Subsequent post hoc Tukey tests were conducted to explore the observed group-level differences, in pairwise comparisons which revealed that Chol-Sulf levels were significantly higher in ACDi than NL (*p* = 0.002), while significant differences were not observed between ICDi and either ACDi (*p* = 0.73) or NL (*p* = 0.23). This pattern therefore suggests that elevated Chol-Sulf levels may be more specific to the allergic subtype.

For Chol-Glc, the lowest mean levels were found in the NL group (0.31 ± 0.30), followed by ACDi (0.63 ± 0.38) and then ICDi (0.87 ± 0.38). The homogeneity of variance was again confirmed by Levene’s test (*p* = 0.092), and the ANOVA yielded a significant group effect (*p* < 0.001) with a large effect size (η^2^ = 0.26); Tukey’s post hoc tests showed that all three groups differed significantly from one another (p_all_ < 0.005). These findings suggest that Chol-Glc may be a sensitive biomarker for both the presence of disease and subtype discrimination, with levels particularly elevated in ICDi.

**Figure 2:**
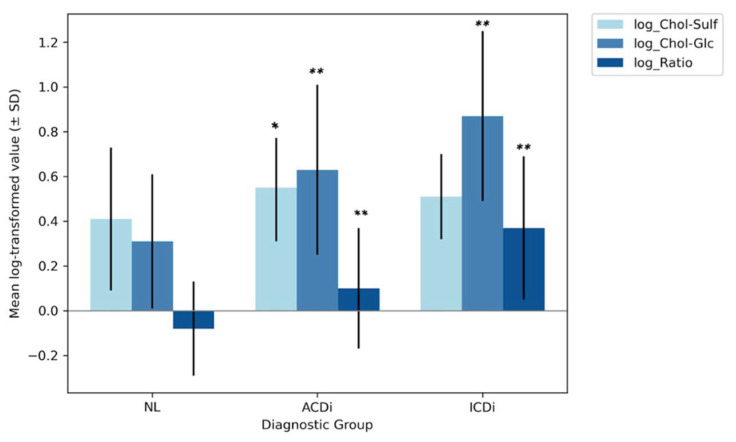
Group-level means of log-transformed biomarkers in non-lesional skin, ACDi and ICDi. Statistical analysis was performed using ANOVA and Tukey’s post hoc tests, resulting in a bar plot displaying mean values (±standard deviations) of log-transformed Cholesterol Sulfate (light blue), Cholesterol Glucosyl (medium blue) and the Chol-Glc/Chol-Sulf ratio (dark blue) across diagnostic groups: non-lesional skin (NL), allergic contact dermatitis (ACDi) and irritant contact dermatitis (ICDi). The x-axis shows the diagnostic groups, while the y-axis represents the log-transformed biomarker levels (unitless), derived from original concentrations in picomoles per milligram (pmol/mg). Each diagnostic group is represented by three bars, one for each biomarker, as well as error bars representing the standard deviation. Asterisks (*) indicate statistically significant differences from the NL group, with * indicating *p* < 0.05 and ** indicating *p* < 0.01.

Lastly, the group mean values for the ratio were again lowest in the NL group (−0.08 ± 0.21), followed by ACDi (0.10 ± 0.27) and then ICDi (0.37 ± 0.32). Levene’s test indicated unequal variances (*p* = 0.015), but, due to the normal distribution of the data and relatively large sample sizes, the use of a standard ANOVA was still deemed appropriate; however, these results should be interpreted with caution. The analysis demonstrated a significant group effect (*p* < 0.001), with a large effect size (η^2^ = 0.27). The subsequent post hoc Tukey tests revealed that all groups differed significantly from each other (p_all_ < 0.001). This therefore suggests that, like Chol-Glc, the ratio may be a sensitive biomarker for both disease and subtype discrimination. The aforementioned ANOVA-derived group-level differences are visually summarized in Figure 2, displaying the mean log-transformed biomarker levels and their SD, per diagnostic group. The numerical results are displayed in Table 1.

### 3.3. Pearson’s Correlation Analysis

**Figure 3:**
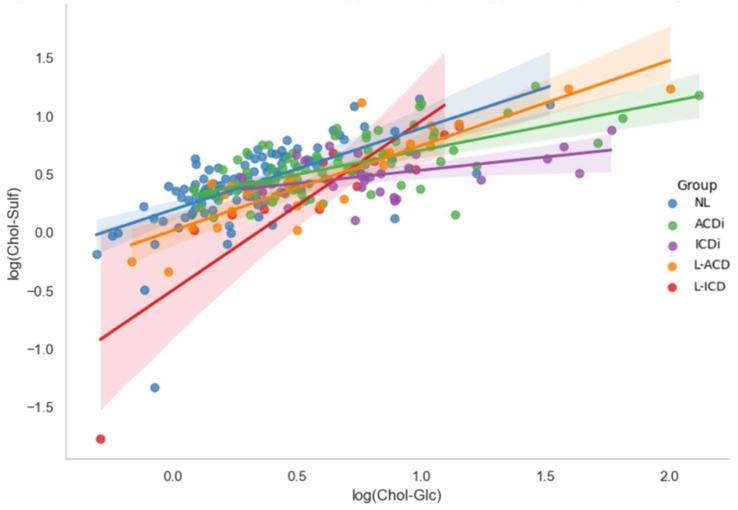
Scatterplot of Pearson’s correlation showing the relationship between log-transformed Chol-Glc and Chol-Sulf across diagnostic groups (NL, ACDi, ICDI, L-ACD and L-ICD). Scatterplot illustrating the Pearson’s correlations between log-transformed Cholesterol Sulfate (log(Chol-Sulf)) and Cholesterol Glucosyl (log(Chol-Glc)) across five diagnostic groups: non-lesional skin (NL), allergic contact dermatitis (ACDi), irritant contact dermatitis (ICDi), lesional allergic contact dermatitis (L-ACD) and lesional irritant contact dermatitis (L-ICD). The x-axis represents Chol-Glc values, while the y-axis represents Chol-Sulf values. These values are unitless due to their log-transformation, where their original values were presented in picomoles per milligram (pmol/mg). Each point represents an individual case, color-coded by diagnostic group. Regression lines with 95% confidence intervals are shown for each subgroup, as to visually reflect the strength and direction of the correlation within these subgroups.

To explore whether the biomarkers provide unique information, their potential co-regulation was assessed via Pearson’s correlation analyses. These analyses were conducted between log-transformed Chol-Glc and Chol-Sulf for the full dataset, as well as for each diagnostic subgroup which is depicted in Figure 3. Across the full dataset (N = 307), a strong positive association was found (r = 0.667, *p* < 0.001), indicating that the biomarkers generally increase and decrease together. This strong correlation between the biomarkers was also seen in the individual analyses of diagnostic groups NL (r = 0.652, *p* < 0.001) and ACDi (r = 0.664, *p* < 0.001), with a more moderate but still significant correlation found in ICDi (r = 0.440, *p* = 0.017). In the chronic subgroups, correlations were found to be even stronger: L-ACD (r = 0.878, *p* < 0.001) and L-ICD (r = 0.859, *p* < 0.001). These results, presented in Table 2, suggest that Chol-Glc and Chol-Sulf are strongly co-regulated, particularly in chronic cases. This co-regulation implies that the biomarkers tend to rise and fall together, which may limit their ability to provide independent diagnostic information when used in combination.

### 3.4. Logistic Regression and ROC Analyses

**Figure 4:**
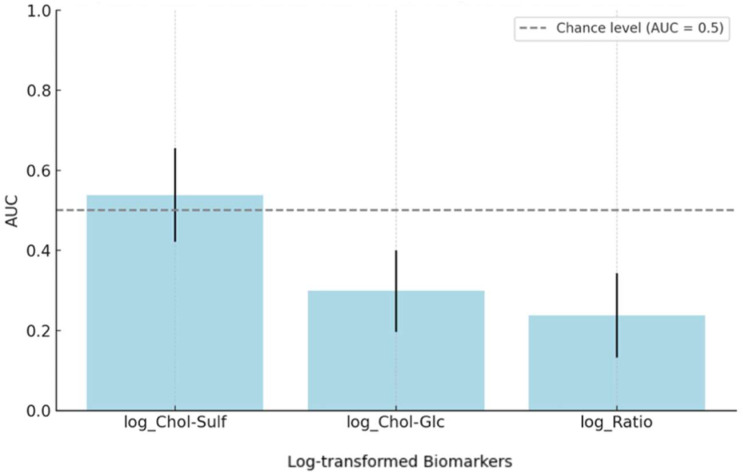
ROC curve analysis: AUC values for biomarker-based classification of ACDi versus ICDi. Bar plot displaying the area under the receiver operating characteristics (ROC) curve (AUC) with 95% confidence intervals for three individual biomarkers: log-transformed Cholesterol Sulfate (log_Chol-Sulf), Cholesterol Glucosyl (log_Chol-Glc) and their ratio (log_Ratio). The AUC values are depicted on the y-axis, reflecting each biomarker’s ability for identifying allergic contact dermatitis (ACDi) versus irritant contact dermatitis (ICDi). An AUC value of 1.0 indicates perfect classification, and 0.5 reflects random chance. The dashed horizontal line represents the chance level (AUC = 0.5). In diagnostic research, AUC values above 0.8 reflect good performance; however, all three biomarkers in this analysis yielded AUCs below 0.8, indicating limited standalone predictive utility for distinguishing ACDi from ICDi.

Finally, logistic regression and ROC analyses were conducted to evaluate the biomarkers’ capacity for distinguishing between ACDi and ICDi. For Chol-Sulf, the regression model was not statistically significant (B = 0.918, SE = 0.993, *p* = 0.355, OR = 2.5, 90% CI [0.49, 12.82]) and the ROC AUC was 0.538 (95% CI [0.421, 0.656]), indicating a poor discriminative ability, as only AUC values > 0.8 are generally deemed clinically useful [29]. Chol-Glc showed a significant inverse association (B = −1.49, SE = 0.55, *p* = 0.006, OR = 0.22, 90% CI [0.09, 0.55]), meaning that higher Chol-Glc levels were associated with lower odds of ACDi. This direction of effect is consistent with earlier group comparisons, which showed higher Chol-Glc levels in ICDi relative to ACDi and NL. However, the model showed a limited predictive performance as well, with an AUC of 0.299 (95% CI [0.197, 0.400]). The strongest statistical effect was found for the ratio marker (B = −2.94, SE = 0.78, *p* < 0.001, OR = 0.05, 90% CI [0.02, 0.19]), again indicating an inverse association with ACDi. However, this marker again showed a low classification accuracy (AUC = 0.238, 95% CI [0.133, 0.343]). These outcomes, summarized in Table 3 and Figure 4, show a significant association but insufficient robust classification for the Chol-Glc and ratio biomarkers when used in isolation.

## 4. Discussion

This exploratory study aimed to investigate whether two cholesterol-derived biomarkers exhibit distinct biomarker profiles in ACD and ICD and whether those profiles would have potential diagnostic relevance. The results demonstrated that there were significant differences in Chol-Glc and the Chol-Glc/Chol-Sulf ratio across all diagnostic groups, suggesting a sensitivity to both the presence and subtype of the disease. However, Chol-Sulf levels did not significantly differ between the diagnostic groups. Despite the promising results for Chol-Glc and the ratio, logistic regression and ROC analyses determined that there was no meaningful standalone predictive power for any of the biomarkers, demonstrated by all AUC values being under 0.6. These results suggest that, although there is important disease-specific patterning of both Chol-Glc and the ratio, all biomarkers lack adequate discriminatory power to serve as independent diagnostic tools.

These results align with the previous research, identifying Chol-Glc as a relevant marker in immune-driven skin inflammation, as Kezic at al. found elevated levels of glucosylated cholesterol in AD [17]. This therefore supports the idea that elevated Chol-Glc reflects immune activation in inflammatory skin conditions, as also observed in both ACD and ICD in this study. In contrast, this study’s findings on Chol-Sulf did not align with the existing literature, such as the study by Fandrei et al., which emphasized the role of Chol-Sulf in barrier structure and lipid fluidity, mechanisms typically disrupted in ICD, indicating that Chol-Sulf levels should be altered in ICD [15]. This was, however, not reflected in the present study, as the only significant difference in Chol-Sulf levels was found in the ACDi vs. NL comparison. This therefore highlights a key distinction between biological relevance and diagnostic utility, as even though both markers are mechanistically involved in skin pathology, only Chol-Glc and the ratio showed subtype-specific patterns relevant for diagnostic purposes. Beyond the acute patch-induced sites, the chronic lesions displayed less distinct biomarker profiles. L-ACD and L-ICD showed increases in Chol-Glc and Chol-Sulf that align with low-grade inflammatory profiles; however, this was very minimal. These findings therefore suggest that biomarker-based differentiation between the CD subtypes may be most useful for acute or early lesions.

Although this study does not present a definitive diagnostic solution, it remains of the utmost importance, as it has identified disease-specific biomarker patterns, thereby warranting further investigations regarding these biomarkers and their potential diagnostic utilization. From a clinical perspective, these biomarkers could be incorporated in addition to, rather than replacing, the current patch testing. Tape stripping of these biomarkers could be rapidly applied during initial consultations and may help prioritize which patients need more extensive patch testing or help support interpretations of patch tests. Thus, the clinical application of these biomarkers could speed up the diagnostic process by avoiding unnecessary patch tests and providing additional information on what is happening in the patient’s skin. Compared with other non-invasive techniques such as TEWL measurement, dermoscopy or optical coherence tomography (OCT), tape-strip biomarkers uniquely provide molecular information directly linked to skin barrier mechanisms at a minimal patient burden, in a timely and low-cost manner [30,31]. Recent advances in lipidomics similarly highlight the potential of non-invasive lipid profiling to detect specific molecular signatures in the skin barrier [32].

To ensure that these interpretations are statistically sound, all statistical analysis methods were validated by UMC statistical analyst Wouter Ouwerkerk, ensuring compliance with the nature of the data and statistical robustness. However, several limitations should be acknowledged, as they may have influenced the observed biomarker patterns. First, an important limitation of this study is the univariate approach used in the regression models, which excluded interactions between biomarkers as well as other relevant clinical parameters which could potentially pose as confounders. Future research should therefore prioritize multivariate biomarker panels that combine these biomarkers, as well as complementary biomarkers such as cytokines or NMF’s, while also taking clinical covariates into account to improve the robustness of the data and the clinical relevance, leading to a more accurate diagnostic performance and translation to real-world patients. Secondly, the allergens and irritants used in the patch tests were grouped by diagnostic category rather than by individual substance. While this approach supported a clearer comparison between the two conditions, it may have masked substance-specific biomarker responses. Moreover, many allergens can possess both allergenic and irritant properties. This overlap could partially explain the biomarkers not being able to clearly distinguish between ACDi and ICDi. Therefore, future research should aim to separate analyses based on the allergen or irritant applied to assess their individual influence on biomarker expression. Thirdly, the grouping of multiple allergens into a single ACDi category was contrasted with a single irritant ICDi group, leading to an asymmetry. This design choice may have impacted internal validity and contributed to the poor discriminative performance observed. Fourth, the analysis was not adjusted to the severity of the patch test reactions, which could also be of influence in biomarker expression, thus possibly distorting the results. Incorporating patch test severity in future analyses would help determine whether the observed differences are due to diagnostic category or reaction intensity. Additionally, this study did not include a proper healthy control group, as the non-lesional samples were taken from people that had been diagnosed with CD, therefore possibly not properly representing truly healthy skin. This should be taken into account when interpreting baseline biomarker levels. Also, Chol-Glc and Chol-Sulf were moderately correlated (r > 0.6), indicating collinearity and thus a limited value in combined models. Finally, the limited sample sizes in the subgroups, particularly ICDi and L-ICD, may limit the statistical power of the analyses and therefore the generalization of the findings that include these subgroups. Increasing the size of and balancing all diagnostic groups would provide a better translation to the real world. These limitations and recommendations should be taken into consideration for further research to enhance the robustness and real-world accuracy of the findings, thereby improving their applicability for future diagnostic implementation.

Altogether, distinguishing ACD from ICD using non-invasive, tape-stripped biomarkers offers a promising first step towards an alternative to traditional patch testing, which is time-consuming, uncomfortable and prone to interpretation bias. While Chol-Glc and the ratio reflect meaningful biological differences between the two subtypes, they are not yet sufficient as independent diagnostic markers. Therefore, their value lies in providing future diagnostic models that combine these biomarkers with additional molecular and clinical data. These diagnostic models may enable a faster, more objective and patient-friendly diagnosis of CD, thereby possibly reducing the duration of the mental and physical burdens of unresolved skin complaints, ultimately improving patient quality of life. Thus, these findings may motivate pilot multivariate models in exploring their diagnostic accuracy.

## Figures and Tables

**Table 1:** Descriptive statistics and ANOVA results for log-transformed biomarkers across diagnostic groups. One-way ANOVAs were performed separately for each biomarker, Cholesterol Sulfate, Cholesterol Glucosyl and the Chol-Glc/Chol-Sulf ratio, to test for overall group differences across diagnostic groups: non-lesional skin (NL), allergic contact dermatitis (ACDi) and irritant contact dermatitis (ICDi). Reported test statistics include mean biomarker levels (M), standard deviations (SDs), F-values, degrees of freedom (F(df)), significance level (*p*-value) and effect sizes (η^2^). This analysis solely included unitless log-transformed biomarker data, with original concentrations expressed in picomoles per milligram (pmol/mg).

Biomarker	Group	Mean (M)	Standard Deviation (SD)	ANOVA F(df)	*p*-Value	Effect Size (η^2^)
log_Chol-Sulf	NL	0.41	0.32	F(2,219) = 6.34	0.002	0.055
log_Chol-Sulf	ACDi	0.55	0.24			
log_Chol-Sulf	ICDi	0.51	0.19			
log_Chol-Glc	NL	0.31	0.3	F(2,219) = 38.87	<0.001	0.260
log_Chol-Glc	ACDi	0.63	0.38			
log_Chol-Glc	ICDi	0.87	0.38			
log_Ratio	NL	−0.08	0.21	F(2,219) = 40.60	<0.001	0.270
log_Ratio	ACDi	0.1	0.27			
log_Ratio	ICDi	0.37	0.32			

**Table 2:** Pearson correlation coefficients across diagnostic groups. Pearson’s correlation coefficients (r) and *p*-values for the correlation between log-transformed Cholesterol Sulfate (log(Chol-Sulf)) and Cholesterol Glucosyl (log(Chol-Glc)), calculated for both the full dataset and per diagnostic subgroup: non-lesional skin (NL), allergic contact dermatitis (ACDi), irritant contact dermatitis (ICDi), lesional allergic contact dermatitis (L-ACD) and lesional irritant contact dermatitis (L-ICD). All correlations proved to be statistically significant (*p* < 0.05), with adequate co-regulation detected within the ICDi subgroup (r = 0.440, *p* = 0.017) and strong correlations for all other diagnostic groups (r > 0.065, *p* < 0.001). The strongest co-regulation was found within the chronic subgroups L-ACD and L-ICD, which suggests that Chol-Glc and Chol-Sulf tend to rise and fall together, especially at later disease stages.

Group	r	*p*-Value
Full sample (N = 307)	0.667	<0.001
NL	0.652	<0.001
ACDi	0.664	<0.001
ICDi	0.44	0.017
L-ACD	0.878	<0.001
L-ICD	0.859	<0.001

**Table 3:** Logistic regression and ROC results of individual biomarkers for the classification of ACDi and ICDi. Binary logistic regression outcomes for the classification of allergic contact dermatitis (ACDi) versus irritant contact dermatitis (ICDi) based on individual, log-transformed biomarkers: Cholesterol Sulfate (log_Chol-Sulf), Cholesterol Glucosyl (log_Chol-Glc) and the Chol-Glc/Chol-Sulf ratio (log_Ratio). Outcomes reported were the regression coefficient (B), standard error (SE), odds ratios (ORs) with 90% confidence intervals (CIs), significance under the threshold (*p*-value < 0.05) and the area under the receiver operating characteristics curve (AUC), with a 95% CI. Observed significant results for both Chol-Glc and the ratio (*p*-value < 0.01) with negative regression coefficients suggest inverse relationships with ACDi diagnosis for both of these biomarkers. However, all three biomarkers showed limited discriminative ability, as demonstrated by low AUC values (AUC_all_ < 0.54).

Biomarker	B	SE	*p*-Value	OR	90% CI	AUC	95% CI
log_Chol-Sulf	0.918	0.993	0.355	2.5	[0.49, 12.82]	0.538	[0.421, 0.656]
log_Chol-Glc	−1.49	0.55	0.006	0.22	[0.09, 0.55]	0.299	[0.197, 0.400]
log_Ratio	−2.94	0.78	0.001	0.05	[0.02, 0.19]	0.238	[0.133, 0.343]

## Data Availability

The data supporting the findings of this study are available on reasonable request from Florentine de Boer and Thomas Rustemeyer (Amsterdam UMC).
